# Health care professional recruitment of patients and family carers to palliative care randomised controlled trials: A qualitative multiple case study

**DOI:** 10.1177/02692163231197917

**Published:** 2023-09-27

**Authors:** Lesley Dunleavy, Nancy Preston, Catherine Walshe

**Affiliations:** International Observatory on End of Life Care, Lancaster University, Lancaster, England, UK

**Keywords:** Palliative care, palliative medicine, terminal care, randomised controlled trial, qualitative research

## Abstract

**Background:**

Trial participant recruitment is an interactional process between health care professionals, patients and carers. Little is known about how clinicians carry out this role in palliative care trials and the reasons why they do or do not recruit participants.

**Aims:**

To explore how clinicians recruit to palliative care trials, why they choose to implement particular recruitment strategies, and the factors that influence their choices.

**Design:**

A qualitative multiple case study of three UK palliative care trials. Data collection included interviews and study documentation. Analysis involved developing and refining theoretical propositions, guided by the ‘6Ps’ of the ‘Social Marketing Mix Framework’ as an a priori framework (identifying participants, product, price, place, promotion and working with partners). Framework Analysis guided within and then cross-case analysis.

**Settings/participants:**

Study investigators and research staff (*n* = 3, 9, 7) from trial coordinating centres and recruitment sites (hospice and hospital).

**Results:**

Cross-case analysis suggests the ‘Social Marketing Mix Framework’ is useful for understanding recruitment processes but wider contextual issues need to be incorporated. These include the ‘emotional labour’ of diagnosing dying and communicating palliative and end-of-life care to potential participants and how the recruitment process is influenced by the power relationships and hierarchies that exist among professional groups. These factors can lead to and support paternalistic practices.

**Conclusions:**

Those planning trials need to ensure that trial recruiters, depending on their experience and trial characteristics, have access to training and support to address the ‘emotional labour’ of recruitment. The type of training required requires further research.

**What is already known about the topic?**

- More high quality trials in palliative care are needed to improve the evidence base that underpins clinical practice but trials can struggle to achieve their recruitment targets.
- Trial recruitment challenges, including clinician gatekeeping, can be amplified in the palliative care context.
- Clinicians have a key role in the recruitment process, but the reasons why they do or do not recruit to palliative care trials is poorly understood.

**What this paper adds**

- The ‘Social Marketing Mix Framework’ is useful for understanding recruitment processes but wider contextual issues need to be incorporated.
- The contextual issues include the ‘emotional labour’ of diagnosing dying, communicating palliative and end-of-life care to potential participants, and the power relationships and hierarchies that exist among professional groups.
- These factors can lead to and support paternalistic recruitment practices.

**Implications for practice, theory or policy**

- Those planning trials need to ensure that trial recruiters have access to training and support to address the ‘emotional labour’ of recruitment.
- Training will be dependent on trial characteristics and clinician experience but may need to include how to discuss a palliative care trial.
- The type of training and support required to address this recruitment barrier requires further research.

## Background

Recruiting sufficient numbers of participants, especially to randomised controlled trials, is a prominent challenge in palliative care research.^1,2^ Trials can be sub-optimal with fewer than 50% of randomised controlled trials achieving their recruitment targets^3^ with reports of palliative care trials only reaching their targets in 37% of cases.^4^ Struggling to achieve statistical power means the trial has the potential to be slow and expensive^5–7^ and more importantly participants will have received an intervention with uncertain benefit and on study completion researchers may still be unable to determine whether the intervention does more good than harm.^3,8^

Why trials struggle to achieve their recruitment targets is complex with some issues not unique to palliative care research such as patient^9,10^ and clinician^11,12^ concerns about randomisation, blinding and placebos. Contextual issues, such as underfunding,^13^ limited infrastructure,^14,15^ high attrition rates^16^ and patient related factors such as a high prevalence of cognitive impairment^17^ contribute to these recruitment challenges in palliative care. Patients are seen as vulnerable especially at the end of life which can lead to clinician gatekeeping.^18^ Gatekeeping is not unique to palliative care research^19^ but is a particularly challenging issue in this population.^20,21^

Clinicians play a key role in the recruitment process, but why they do or do not recruit to palliative care trials is poorly understood. Recruitment is not a single event and is often a lengthy and complex process typically involving three steps; identifying, approaching and consenting.^22^ It occurs in real time, in real clinical settings and it can be a difficult activity as it disrupts the usual clinician/patient relationship.^23^

What strategies may facilitate trial recruitment^3,24^ or to research studies in general^22^ is hampered by a lack of high-quality evidence. A review exploring recruitment barriers and facilitators in palliative care trials using the ‘6 Ps’ of the ‘Social Marketing Mix Framework’ as a theoretical framework was conducted.^25^ The ‘6 Ps’ are; identifying participants, product, price, place, promotion and working with partners (see Table 1 for definitions).^26^ The review found that the evidence underpinning the barriers and facilitators to palliative care trial recruitment was largely anecdotal and more methodological research was needed. It suggested the ‘6 Ps’ may help researchers better understand recruitment processes but further exploration was required.^25^

**Table 1.** The ‘6 Ps’ of the ‘Social Marketing Mix Framework’.^26^

Elements	Definitions
Identifying participants	Defining the target audience.
Product	
Defining the product	The intervention is the product (its scientific, theoretical basis, does it meet the needs of the target audience?), the product must address a problem that is perceived as serious and amenable to the intervention.
The product’s competition	The amount of competition for the participant’s time and energy.
Price	The cost to the potential participant of taking part in the study (e.g. financial, time, physical and emotional effort). Things need to consider: type of costs and how to minimise the costs.
Place (improving accessibility)	‘The location where the participant will receive information about, or engage in, the intervention’.
Promoting the study	‘Identify the acceptable avenues that reach the target population’.
Working with partners	‘Partners are defined as organisations involved with a social change effort or serving as conduits to target audiences’. Things to consider: partner education, partner referrals and recruitment and barriers to partnering.

This study aims to address this gap in knowledge by exploring how clinicians recruit patients and carers to palliative care trials, why they choose to implement particular recruitment strategies, and the factors that influence the decisions they make.

## Methods

### Research question

*How do health care professionals recruit patients and their family carers to palliative care randomised controlled trials and why do they use certain strategies during the recruitment process?*

### Design

A retrospective, descriptive qualitative multiple case study was conducted following Yin’s^27^ approach with a critical realist lens^28^ being applied throughout the study (further details below).

### Case definition

In this study cases were defined as; UK palliative care randomised controlled trials aimed at adult patients with incurable cancer and/or advanced, progressive non-malignant disease and their family carers; and either the primary endpoint was symptom control and/or quality of life; or it tested an intervention that was clearly a palliative care intervention and the study primary endpoint was survival (further details in Table 2).

**Table 2.** Case and participant inclusion criteria.

Case inclusion criteria
• Palliative care randomised controlled trials aimed at adult patients with incurable cancer and/or advanced, progressive non-malignant disease and their family carers will be included. and either
• Palliative care randomised controlled trials where the primary endpoint is symptom control and/or quality of life will be included. or
• Palliative care randomised controlled trials that test an intervention that is clearly a palliative care intervention and the study primary endpoint is survival will be included.
• UK trials registered on relevant trial registers and databases. These databases were the National Institute for Health Research Portfolio database (renamed as UK Clinical Trials Gateway during the study), the International Standard Randomised Controlled Trial Number (ISCRTN) registry, Cancer Research UK and Clinical Trials.gov.
• Trials that are either ongoing, recently closed (within 12 months) or set up during the data collection period.
• Trials that have been open for at least four to six months to ensure enough time for a recruitment plan to have been trialled, assessed and changes implemented if required.
• For trials that are closed, this will need to have happened within the previous twelve months to ensure participants are able to recall their experiences of recruiting to the trials.
Participant inclusion criteria
• Staff involved in the recruitment of patients or carers in the selected ‘cases’ from the study coordinating centre and clinical recruitment centres will be included such as the Chief Investigator, Trial Manager, Clinical Research Associate, Principal Investigator, Research Nurse or other clinicians.
• 18 years of age or over.
• Be able to read and communicate in English.

### Case selection

Cases were consecutively screened and identified by LD in consultation with CW and NP from the trial databases listed in Table 2. There were 18 eligible trials available at the time of screening (from 11/2016 to 10/2017). Cases were chosen that had a variety of study designs and were recruiting in different settings to reflect theoretical replication. Yin argues that study findings are more robust if cases corroborate each other despite having contrasting characteristics.^27^ Practical issues also influenced case selection such as the number of eligible trials available in the UK at the time of sampling and a small number of trials could not be included because research team members were involved in the trial.

### Case recruitment

The Chief Investigators of the three selected cases were approached by email and asked if they agreed for their trial to be a case.

### Participant definition

Professionals with different recruitment roles, from both the study coordinating centre and clinical recruitment centres within each case, were eligible (see Table 2). A study coordinating centre was defined as overseeing the conduct of a trial^29^ while a recruitment centre was a clinical setting where recruitment activity took place.^30^

### Participant recruitment

The Chief Investigator of the selected cases circulated study information to eligible participants. Participants were then asked to suggest other individuals with relevant experience. Recruitment ceased within the case when the pool of eligible participants who were willing to participate was exhausted. Participants provided verbal consent.

### Theoretical propositions

Initial theoretical propositions (predicted theory about what may be learned from examining the case) were developed to guide data collection and analysis, influenced by the ‘Social Marketing Mix Framework’,^26^ literature review findings^25^ and wider trial recruitment literature. Yin recommends their use to explore the deeper reasons for what can be observed, reflecting a critical realist stance, and also so study findings can be generalised beyond the ‘cases’ through ‘analytical generalisation’.^27^ Yin provides little practical guidance on how theoretical propositions work alongside an a priori theoretical framework, as in this study. This influenced the decision to develop only a small number of theoretical propositions relating to only three of the ‘Ps’ in the ‘Social Marketing Mix framework’. Product, working with partners and place were specifically chosen because of literature review findings and issues raised within the general trial literature. For example, being able to access dedicated research staff was the strategy most discussed in the literature review.^25^

They were;

- Study design influences how recruiting staff undertake the process of recruitment and the strategies they use. (Product)
- The involvement of specific research staff in the recruitment process impacts on how well the trial meets its recruitment target. (Working with Partners)
- How recruiting staff undertake the recruitment of patients or carers is influenced by their professional role. (Working with Partners)
- Where recruitment activity takes place may influence the recruitment process. (Place)

### Data collection

Data were collected from each case consecutively. A telephone interview was conducted with each participant to capture multiple perspectives of the recruitment process and trial documentation was collated to understand how these processes were formally communicated.^27,31^ Online trial documents available in the public domain were identified and participants were asked to suggest documents. The interview topic guide was iteratively developed throughout the study and covered; recruitment procedures, exploration of phraseology used to discuss the trial with participants, how well the trial had recruited, factors that helped or hindered recruitment, recruitment strategies and lessons learnt (see Supplemental Material). LD, a nurse researcher with specialist palliative care clinical and research experience, conducted the interviews for her PhD. Participants were not known to LD. Interviews were audio recorded, anonymised, transcribed and field notes were made.

### Data analysis

Data analysis was an iterative process with framework thematic analysis^32^ being used to compare and contrast data within and then across cases to identify patterns. This approach involves five interconnected stages: familiarisation, identifying a thematic framework, indexing, charting, and mapping and interpretation.^32^. The ‘6 Ps’ were used as the a priori analytical framework. Raw data was reviewed, labelled, sorted and synthesised; descriptive accounts were developed by identifying key dimensions/elements, refining categories and developing classifications and finally explanations were developed to account for the data. Analysis was carried out by LD with critical input from CW and NP.

### Research ethics and approvals

Approval was obtained from Lancaster University Faculty of Health and Medicine Research Ethics Committee (Reference number: FHMREC15042, 22nd February 2016). The study is reported in line with the DESCARTE checklist.^33^ Reflexivity was considered throughout the study,^34^ including regular supervisor meetings, to try and limit subjectivity and bias as recommended by Yin.^27^ Participation was voluntary and anonymisation was assured.

### Findings

Data collection occurred between March 2017 and June 2018. Three cases were included and 19 participants (*n* = 3, 9, 7) took part with the mean interview length being 39 min (range 25–60 min). Study findings are presented as a cross case analysis with data from each of the cases dispersed throughout (see Table 3 for case and participant characteristics).

**Table 3.** Case and participant characteristics and trial documentation collected and analysed.

	Case one	Case two	Case three
Recruitment setting	Hospice inpatients	Hospital outpatients	Hospital inpatients
Trial population	Advanced cancer patients	Advanced cancer patients and their carers	Advanced cancer and non-cancer patients (or proxy if required)
Trial design	Non-pharmaceutical placebo trial	Parallel trial of a complex non-pharmaceutical intervention	Feasibility cluster trial of a complex non-pharmaceutical intervention
Recruitment target	The recruitment rate was described as slow and at the time of data collection, approximately 83% of the recruitment target had been met. This had taken a number of years to achieve and took longer than anticipated.	Achieved over 30 months rather than the anticipated 24 months	Only two of the four sites reached their recruitment target with recruitment taking longer than the anticipated three months. One of the sites (intervention) took 6 months to reach its recruitment target while the other (control) took four and a half months
Number of interviews	3 interviews	9 interviews	7 interviews
Type of participants	Palliative medicine consultant = 1 Research nurse = 2	Hospital consultant = 2 Specialist nurse = 2 Research nurse = 5	Senior academic = 2 Researcher = 1 Palliative medicine consultant = 1 Research nurse = 3
Type of documentation collected and analysed	Study protocol, patient information sheet, patient consent form, GP letter, UK Clinical Trials Gateway website, results paper.	Study protocol, patient information sheet, patient consent form, carer information sheet, carer consent form, carer GP letter, patient study recruitment poster, trial recruitment figures for each hospital site, monthly recruitment figures for site four, an invitation to participate in the trial for clinical recruitment centres, ‘Frequently asked questions’ document for health care professionals, published protocol, published results papers, UK Clinical Trials Gateway website	Study protocol, patient information sheet (intervention and control), patient consent form, carer information sheet (intervention and control), carer consent form, consultee information sheet (control and intervention), consultee approval form for continued participation if capacity is lost, recruitment letter to bereaved relative, trial recruitment figures for each site, clinical scenarios and materials to support recruitment for health care professionals, published study conference posters, published results papers, UK Clinical Trials Gateway website

**Figure 1.**
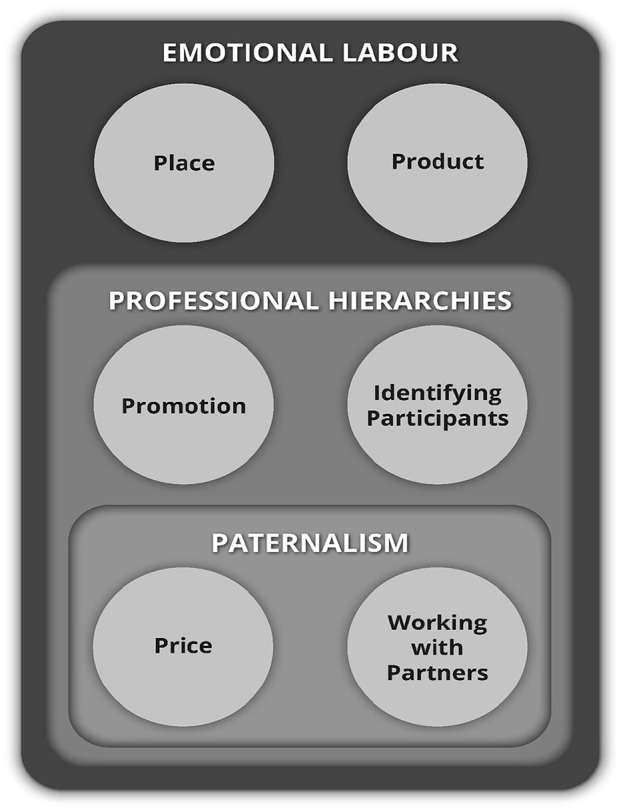
New palliative care trial recruitment framework proposed.

First, the findings are presented in relation to the ‘6 Ps’, the a priori framework, reordered to reflect the findings. These are summarised in Table 4. Second, three overarching classifications are presented that were derived interpretively from the data; ‘emotional labour’, ‘paternalism’ and ‘professional hierarchies and power relationships between clinicians’. How these map onto the ‘Social Marketing Mix Framework’ is presented in Figure 1.

**Table 4.** Summary of findings in relation to the ‘6 Ps’, the a priori framework.

	Summary of findings	Illustrative quote
Working with partners: partner referrals and recruitment	• Clinician interest in the trial was not enough to guarantee suitable recruitment centres were enrolled into the study. • Across the cases, potential participants were identified in routine multidisciplinary team meetings and during informal discussions between clinicians with research nurses reviewing clinic lists and medical notes to confirm eligibility.	‘The other thing then was to vet every centre as we did . . ., to make sure that they passed the test and that they’ve got a recruitment record and that we didn’t have to invest a great deal of resource to set them up in order to do the trial, ‘cos there’s no point having somebody with interest but no access to research method’. (*Chief Investigator, case two*)
Identifying participants: defining the target audience	• Predicting prognosis was a requirement when determining eligibility. In cases one and three, clinicians needed to estimate this while in case two a performance status scale was used. Applying subjective eligibility criteria proved particularly problematic in case three, especially in the control arm, as doctors were required to predict the patient’s risk of dying during admission. Supporting and training staff to do this could potentially lead to contamination.	‘Without this level of education and support there is wide variability on the interpretation of this criterion with tendency for prognostication rather than consideration of risk. However, providing this level of education and support would result in contamination in the control sites’. (*Protocol, case three*)
Product: The product’s competition	• A notable recruitment barrier in case two was competition from treatment trials with specialist centres running multiple trials with similar eligibility criteria. Recruiting staff prioritised these and because of funding would consider prioritising trials that were struggling to meet their target or were going to close soon. • Additionally, starting chemotherapy prior to consent was an exclusion criteria which could ‘make it tricky’ (*Research nurse five, case two*) to recruit patients. Patients were sometimes keen to start treatment and did not want to consider a palliative care trial.	‘. . . we get sort of paid as a Trust [hospital] and a research department for meeting those targets that we set. And so we sort of are constantly aware I guess that we have a certain number of patients to get into each trial, so I don’t think as a team that is at all at the forefront of any decisions that are made but potentially for trials that aren’t recruiting so well, and if someone was eligible for a few different trials that were not for the same thing, and equally could benefit that patient then I personally think sometimes we might lean towards the one that was maybe not recruiting so well you know to help with numbers’. (*Research nurse five, case two*)
Product: Defining the product	• Recruiting staff described why they thought participants were interested in the trial and reasons included not being a drug trial (case one) and receiving research nurse support even in the control arm (case two). • Research nurses (case two) also described why they thought some patients were not interested in the trial and reasons included not being a treatment trial; extra hospital visits, not interested in any trial and the intervention was not needed. • Patients were not always in clinical equipoise and could have preconceived ideas about which trial arm they wanted to be allocated to which could influence their level of interest in the trial.	‘Some patients they will say to you, do you know what, no offence but I don’t want to be coming into hospital regularly, I just want to go and live my life and I’m feeling good at the moment and no thank you, I don’t want to participate and that’s absolutely fine, whereas others do want that extra support I guess and knowing that they have the research team available to them helps I think’. (*Research nurse one, case two*)
Price: ‘Type of costs’ and ‘Minimising the costs’	• Recruiting staff (cases one and three) felt the patient’s unstable and fluctuating condition influenced their ability to engage in the recruitment process. Fatigue and symptom burden were issues so research nurses would go through the study information to try and minimise burden. This was important to increase trial acceptability. • Taking on the role of consultee (someone to advise on what the participant’s wishes would be if they were able to consent for themselves, and on whether they should take part in the study) could be too burdensome for some carers in case three because of competing demands on their time. • Across all cases, recruiting staff also felt the patient’s psychological and emotional well-being impacted their ability to engage in the recruitment process. Patients were perceived as not always being ‘in the right frame of mind’ (Chief Investigator, case one) due to just being diagnosed, living with uncertainty or requiring hospice care. Recruiting staff wanted to minimise study burden by introducing them to the study at an ‘appropriate’ (Research nurse five, case two) time.	‘. . . a relative would be like we’re quite busy with some other paperwork and we don’t know what’s going on, we need to get some you know equipment in place before discharge, so it’s most of the time they people do not have a problem about the topic of research, but it’s mostly about not having time or not having the energy to complete the questionnaires or even go through the consent process really’. (*Researcher, case three*)
Place	• In case two, nurses felt some patients were discouraged from participating because of parking issues and the need to travel to the recruitment centre despite costs being covered. • The importance of understanding the patient’s care pathway when estimating a centre’s (specialist and non-specialist hospitals) eligibility rates was highlighted in cases two and three.	‘so even though they said we see lots of (name of diagnosis), actually they only saw them for the diagnostic bit, when it came to the care, he was transferred to another local hospital . . . So what we did was open a centre where they were sending the patients out to, so recognising the patient pathway . . .’ (*Chief investigator, case two*)
Working with partners: barriers to partnering	• Across cases, clinicians acted as gatekeepers. Research nurses accepted the opinion of other clinicians that it was not appropriate to approach certain patients but in case one this did not always happen. Their decision was based on how much they trusted the opinion of the staff member and sometimes sought a second opinion from the Chief Investigator. • Lack of clinician engagement made addressing gatekeeping challenging especially in the inpatient setting. It was difficult to engage doctors because of staff rotation and turnover and time pressures. Some had a limited knowledge of research or did not see it as part of their role or routine care. Identifying and utilising the support of staff who were the most engaged was a useful strategy. • Across all cases, resource issues impacted the time recruiting staff could work on the trial. Working within a voluntary organisation was challenging because of limited funding and research infrastructure.	‘. . . but I often get a second opinion with other because there are certain members of staff that I know don’t want to and I have tried, you know I have really tried to engage and I have done little sessions on research and why we do it and you know the importance of progress and so on. Umm but yes that’s I say that’s quite high up on what I find most challenging about my job really’. (*Research nurse one, case one*) ‘I think that you learn to fight your battles. I think that you know who you can approach, and who is less open to research, within staff’. (*Research nurse two, case one*)
Promoting the study	• Roles and responsibilities were determined by professional role with the initial approach usually made by the doctor in charge of the patient’s care but specialist nurses also took on this role in case two. Research nurses would not approach the patient before seeking medical permission. • Across cases, recruiting staff needed to be flexible and demonstrate respectful persistence. Nurses felt building trust and rapport with potential participants was important and carer engagement was a way of achieving this. Unsurprisingly, having had previous contact made this process more straightforward. • Recruiting staff needed to pay attention to key and careful messaging when introducing the trial. Nurses felt randomisation was a difficult concept for patients to understand in all trials and not just in palliative care. • Palliative and end-of-life care needed to be discussed during informed consent (cases two and three) because of the intervention type, participant information and setting. Palliative care could often be associated with end-of-life care and in case two was removed from the study title to shift the focus to symptom control as ‘we thought that was a better sell, to try and get older patients in’ (Chief Investigator, case two). In order to demystify the terms and to increase trial acceptability, recruiting staff would explain palliative care in terms of symptom control and extra support.	‘And, you’re very flexible, because, again, with palliative patients, you may plan to go and see somebody at a certain time, and they’re ill, or they’re on the toilet, or their family’s come, so you really do need that, sort of, flexibility’. (*Research nurse two, case one*) ‘Well I think the patients that I recruited I already knew, and so it was easy for me to go to them ‘cos I’d been involved in some prostate studies and the patients that I recruited were those patients that had become end of life, so my approach was very much you know we already had a relationship’. *(Research nurse three, case three)*
Working with partners: partner education	• Previous clinical experience influenced how much preparation and training research nurses needed to work on a palliative care trial. • Study coordinating centres maintained regular contact with recruiting staff to promote engagement using various strategies that included newsletters, site visits, teleconferences and clinician incentives. • Research nurses felt it was crucial to personally engage and maintain engagement with clinicians to address gatekeeping and the strategies they used included; one to one contact, presentations and attending staff meetings. • Across cases, having a ‘research champion’ on site was important to promote the trial and to engage with other clinicians. A role largely carried out by the lead medical clinician but specialist palliative care professionals also took on the role (case three). This was felt to have a detrimental impact on recruitment, as they were often unavailable due to be being a consulting service.	‘. . . we had two sites where the PIs were absolutely on board with it, this is important, we’re committed to doing this, we’re going to do it, and they were both sort of positioned within a functioning clinical team, so that was one control and one intervention. And they were basically able to sort of lead their team, we’re doing this study, this is important, and then got everybody on board and committed and worked with the research nurses . . .’ (*Chief Investigator two, case three*)

### The ‘emotional labour’ of recruiting patients and carers to palliative care randomised controlled trials

‘Emotional labour’ involves the management and regulation of feelings in the workplace and is broadly defined as ‘the induction or suppression of feeling in order to sustain an outward appearance that produces in others a sense of being cared for in a convivial safe place’ (p. 11).^35^ Across cases, recruiting staff had to manage the ‘emotional labour’ of approaching participants at a difficult time in their illness trajectory and they had to respond rapidly due to the risk of deterioration or start of treatment.

#### Costs for research nurses

The ‘Social Marketing Mix Framework’ does not acknowledge the ‘Price’ or ‘emotional labour’ of recruiting to a palliative care trial for research nurses, as ‘Price’ applies to patients and carers. Balancing the need to respond rapidly while taking account of the patient’s unstable condition was an issue in cases one and three which could be exacerbated by wider resource issues. Recruitment could be time consuming because of the need for multiple visits and the support patients required to go through study information:

. . . you know they’ve said, you know I can’t actually, I’ve not felt up to reading it or you know I can’t read the information, you know so I have sort of read it to them and then gone through it with them so things just take a bit longer and you need oodles of sort of patience and also not giving up really. . . (*Research nurse one, case one*)

In case three, complex consent processes proved particularly challenging for some research nurses as they were required to seek consultee assent or if the patient had capacity, follow advance consent procedures. They could lack confidence and skill when assessing capacity and could be unclear about who could act as consultee. Lack of experience and training contributed to this recruitment barrier with the Chief Investigator reflecting on how they could have prepared the nurses better.

Multiple carer visits were required with some wanting to discuss it with other family members before making a decision. Allowing carers the time they needed while balancing the pressure to recruit within a short window of opportunity could lead the research nurses to face a difficult dilemma and experience increased emotional burden:

. . . but I can’t say to somebody I know you want to discuss it with your brother but your mum’s only eligible right now and she could change tomorrow, do you know what I mean, I can’t say that to them. I just have to go that’s fine, they can discuss it with whoever they want, you know they can take as long as they want . . . (*Research nurse one, case three*)

#### Choosing the best time to approach patients

Across all cases, recruiting staff made judgements about when to introduce the trial based on their concerns about the patient’s physical condition, psychological and emotional wellbeing and practical considerations such as visiting times. They were concerned that patients were ‘overwhelmed’ (Research nurse one, case two) with information and so to increase trial acceptance nurses allowed patients time to process or digest what was happening to them before introducing the study, an approach some expressed they also used in non-palliative care trials. This approach also appeared beneficial for recruiting staff particularly the research nurses:

People’s understanding of hospices, tends to be that it is a place you go to to die. So, when people are referred to the in-patient unit, and they’re admitted, there is that, sort of, automatic barrier comes up, so, oh my God, is this it? So, it’s, from my perspective, really helpful not to see somebody within the first 48 hours. Because, they need to gain a confidence in what we are doing, as an organisation, that the place is, you know, a good place to be, and they need to, sort of, feel safe and secure. (*Research nurse two, case one*)

When introducing the study, research nurses would vary the amount of information given depending on the patient’s response, level of understanding and their own level of comfort. Recruiting staff felt patients responded to being approached about a palliative care trial differently. Some were very accepting and viewed it positively while others were less open. Some were not ready to acknowledge or talk about their illness and a nurse described how a minority became distressed:

. . . and then I had a couple of patients who actually got really angry in that leave my room, I don’t want to talk to you, I’m going to get better, how dare you start talking about such, you know, that I’m not going to get better, that’s negative thoughts, I don’t need that, go away, you know sort of real mixed kind of responses, sort of total denial so the patient had been told . . . (*Research nurse one, case three*)

Introducing the study appeared less demanding for those nurses who had had previous clinical contact with the patients as they could strike up an instant rapport and they knew what the patient understood about their condition.

### Explaining palliative care

Recruiting staff (cases two and three) could find explaining palliative care challenging as it was difficult to discuss without acknowledging its association with end-of-life care. Research nurses had to broach issues that they would not routinely have to discuss which could cause worry and anxiety:

. . . I actually think I made a mountain out of a molehill ‘cos actually I think the patients were quite fine about it, I think it was a lot of it was our worry about how they would feel, because we’d kind of never it was quite new to us . . . (*Research nurse three, case two*)

In case two, research nurses felt uncomfortable discussing a bereavement questionnaire as part of the consent process because they were apprehensive about how participants may react and how it may lead to difficult questions which they felt they did not have the skills to answer. The importance of highlighting the questionnaire to limit the potential for distress was discussed:

. . . I had that discussion she burst into tears, so it wasn’t ideal, and she said to me afterwards she was like I’m really pleased that you highlighted that from the information because I wouldn’t have wanted to have been at home and burst into tears having read this, but she said that’s really upsetting and she sort of said I can see why you want to do it and it makes sense . . . (*Research nurse five, case two*)

#### Preparation for a sensitive conversation

Across all cases, those research nurses who had experience of talking to palliative care patients and carers appeared to find having sensitive conversations less emotionally demanding. Discomfort and inexperience manifested itself in staff declining to work on case three with centres having to use a core team of nurses who were comfortable working on the study:

And the nature of the patients, some of our team didn’t like approaching them because they weren’t used to that type of patient, that was a problem as well. (*Research nurse two, case three*)

Despite previous experience, there were still concerns around how to broach conversations around death with participants in cases two and three. Research nurses prepared for sensitive discussions by discussing how best to approach these conversations within their own team and by seeking advice from the palliative care team. Working within a voluntary organisation as a research nurse, where resources are limited, could be challenging:

. . . working on your own can be quite difficult and I’ve always been part of a research team so you know in my last job, . . . you’ve always got those colleagues that are working in exactly the same way as you that you can run things by or umm you know I’m very, I’m quite isolated really in my job and at times it can really upsetting and stressful and challenging and I don’t always feel that I really have anyone to share that with. (*Research nurse one, case one*)

### The influence of ‘paternalism’ on palliative care trial recruitment

Paternalism refers to; ‘the intentional overriding of one person’s preferences or actions by another person, where the person who overrides justifies this action by appeal to the goal of benefitting or of preventing or mitigating harm to the person whose preferences or actions are overridden’ (p. 215).^36^ There was evidence that clinician and carer paternalism could override patient autonomy in the trial recruitment process.

When screening, clinicians applied their own unofficial eligibility criteria and in case two, believing they were acting in the patient’s best interest, information was withheld from patients about all the trials they were eligible for. Treatment trials were seen as having the potential to ‘actually benefit (the patient) clinically’ (*Research nurse three, case two*) so were prioritised.

Carer gatekeeping was a particular issue in case three where patients were at risk of dying. Nurses sometimes needed to introduce themselves to the carer before or at the same time as speaking to the patient. A nurse described how she was fearful of approaching patients, as she was worried about the carer’s reaction, including concerns they may make a complaint. Families could became annoyed and it could be a dilemma balancing the right to approach a patient with managing the carer’s distress:

I literally couldn’t even tell them who I was and what I was doing or finish the sentence before they were like now’s not the time, how dare you? Well it’s just we do have to ask at such a difficult time because of the timing of the research, I appreciate it’s difficult but the timing is necessary. But it’s not appropriate, they just weren’t listening. (*Research nurse one, case three*)

### The influence of ‘professional hierarchies and power relationships between clinicians’ on recruitment practices

Professional hierarchies and power relationships influenced the recruitment of clinical recruitment centres, trial participants and the research champion within the research site. Chief Investigators who were also senior doctors used their medical contacts to identify research sites while the Chief Investigator from an academic background needed to negotiate access by building up relationships with clinicians which could take a long time:

. . . So it was really just a question of convincing them and just negotiating with them and building levels of rapport which I think were really really critical. It’s not just a question of parachuting into a site, there’s so much ground work that needs to take place beforehand. (*Chief Investigator one, case three*)

Any care team member could identify participants but confirmation of trial eligibility was the responsibility of the lead medical clinician as they held overall ownership of the patient’s care. Medical confirmation that the patient was a palliative or end-of-life care patient was required. Research nurses used multi-disciplinary team meetings to identify eligible patients and to seek medical confirmation of eligibility. These meetings could be used as a forum for doctors to decide who the nurses could and could not approach:

So if they were somebody that was likely to be fit enough for a chemotherapy trial, then we would get them to see the medical oncologist and the nurse specialist would not talk to them about (name of trial) . . . if we felt that they were not really suitable for either then we would then get the nurse specialist to discuss with them . . . (*Doctor, case two*)

Confirming eligibility was more problematic in case three as some doctors appeared reticent and fearful of making the decision that the patient may die under their care and how it could be difficult for recruiting staff to ‘get past people’s inherent optimism’ (Doctor, case three). In order to confirm eligibility a difficult conversation with the patient needed to occur and research nurses wanted to make sure this had happened before approaching but conversations could be poorly documented. They needed to see written confirmation that it was safe for them to approach the patient because of the sensitive nature of the information they needed to present. Similar concerns were raised by a palliative medicine doctor:

. . .we couldn’t go to people who didn’t know, weren’t aware obviously that would come as a shock, perhaps they had been told but then forgotten, or that we were concerned that if they read that they you know they might not have realised . . . (*Doctor, case three*)

Research nurses also had the power to influence whether or not a potential participant was recruited as they felt they were ‘the patient’s advocate’ (Research nurse three, case two) and their role was to act in the patient’s best interest.

The requirement for the lead medical clinician to confirm eligibility influenced who was the most appropriate professional to act as ‘research champion’. In practice, who took on the role of Principal Investigator was often a practical decision, based on their enthusiasm. In case two, this role was carried out successfully by a specialist nurse reflecting their position within the multi-disciplinary team:

. . . the MDT team makes a decision, the news is broken to the patient, and then ordinarily there’s like a small break out room where the patients can you know deal with . . . their emotions, and then the specialist nurses had a choice basically between either whether the patients were receptive at that stage to learn about the trial . . . (*Chief Investigator, case two*)

Research nurses valued the time specialist nurses spent talking to patients about their illness as it freed them up to focus on the more practical aspects of the recruitment process.

Figure 1 illustrates the new palliative care trial recruitment framework proposed that reflects the study findings. An adapted ‘Social Marketing Mix Framework’ that incorporates the wider overarching contextual issues of ‘emotional labour’, ‘paternalism’ and ‘professional hierarchies and power relationships between clinicians’.

## Discussion

### Main findings

The cross-case analysis suggests the ‘6 Ps’ are relevant in the context of palliative care trial recruitment but the concepts of ‘emotional labour’, ‘paternalism’ and ‘professional hierarchies and power relationships between clinicians’ are required as an additional theoretical lens. This is to better understand how health care professionals recruit patients and their family carers to palliative care trials and why they use certain strategies during the recruitment process. Clinicians experienced ‘emotional labour’ when they were ‘promoting’ and recruiting to a palliative care trial which lead to paternalistic recruitment practices. The ‘professional hierarchies and power relationships’ that existed between clinicians influenced how ‘emotional labour’ was experienced by medical and nursing staff and also facilitated and supported paternalistic recruitment practices. The final theoretical propositions that reflect the emerging findings and the wider literature are outlined in Table 5.

**Table 5.** Final theoretical propositions.

• The use of subjective criteria to predict a patient’s prognosis as part of a palliative care trial’s eligibility criteria acts as a barrier to recruitment.
• Involving recruiting staff who have previous experience of caring for palliative care patients and their carers will be a facilitator to recruitment.
• The provision of training for recruiting staff on how to introduce a palliative care trial to patients and carers will help address health care professional gatekeeping.
• The provision of ongoing support for those involved in recruiting to a palliative care trial will help address health care professional gatekeeping.
• Choosing a Principal Investigator who has overall responsibility for the patient’s care influences how well the trial meets its recruitment target.

### What this study adds

The ‘emotional labour’ of trial recruitment is not new^11,37^ and unsurprisingly palliative care contextual factors contributed to this issue in this study. The ‘emotional labour’ of caring for the dying especially in the hospital setting has been recognised.^35,38^ Palliative care is carried out in an emotion laden context with clinicians often having to deal with appropriate but powerful patient and carer emotions.^39^ Patients and carers often associate palliative care with death and dying^40,41^ and clinicians can find initiating end of life conversations challenging and can feel ill prepared.^42,43^ These issues are problematic in the recruitment context as an ‘active’ rather than a ‘passive’ communication stance is required.^44^

Tailoring information according to levels of understanding and readiness is viewed as paramount in end-of-life communication^45,46^ but this can be difficult for those going through standardised participant information. Fluctuating levels of patient and carer awareness can also make it challenging for recruiting staff to ‘promote’ a palliative care trial. Respecting ‘awareness’ preferences is important^47^ but must not be used by clinicians as a reason to avoid research discussions.

Doctors are viewed as largely being responsible for discussing prognosis and making treatment decisions^48,49^ but they can struggle to recognise when a patient is approaching the end of their life.^50^ The ‘emotional labour’ of predicting a patient’s prognosis has been identified as a reason for doctors avoiding end-of-life care discussions^43^ so according to Glaser and Strauss^51^ perpetuating ‘closed awareness’. Closed awareness is when only clinicians and families are aware that the patient’s illness will lead to their death.^51^ This has implications for the recruitment process as the lack of prognostic certainty can lead to a reluctance to confirm eligibility and ‘promote’ the trial to participants. There can also be tensions between consulting specialist palliative care services and those that focus on a more acute model of care.^47^

Ethics committees can be concerned about involving patients and family carers in palliative and end of life care research. They can be fearful of overburdening vulnerable patients^21^ and may insist on a clinician acting as a gatekeeper in the belief this will protect patients and reduce the risk of distress. This study has shown that health care professionals may have complex reasons for not approaching palliative care patients about research. This has important implications for the recruitment process as ethics committees may be inadvertently creating a barrier to participant enrolment by requiring clinicians to introduce a research study to the patient.

This study has highlighted the importance of incorporating ‘partner education’ into trial planning for recruiting staff to manage their emotional labour, including how to discuss a palliative care trial, echoing one of the recommendations from the updated MORECare project.^52^ This is in addition to the generic trial training recommended in the general literature to improve recruitment rates.^37,53^ Training should reflect trial characteristics and clinician experience and may need to include how to; explain palliative and end-of-life care^54^; assess the participants understanding of their condition^55^; manage the psychological needs of patients and carers^56,57^; and assess capacity and enact proxy and advance consent procedures.^52,58^ Staff should also have the opportunity to reflect on their practice.^59^ Integrating research into routine practice^60^ will mean clinicians already have a rapport with participants.

### Strengths and weaknesses of the study

To the author’s knowledge, this is the first qualitative multiple case study to produce new insights into the palliative care trial recruitment process. By using theoretical propositions and multiple diverse cases the findings can be used to understand trial recruitment processes beyond the three cases^27^ and are relevant to palliative care research generally. An additional case would have been selected if resources had allowed as findings suggested pharmaceutical symptom control trials may raise particular issues for staff. The views of participants who agreed to take part may not be representative of others involved in the recruitment process. Patient and carer perspectives are not captured and they may be different to those expressed by clinicians.^61^ This study took place in the UK and internationally communication preferences,^62^ research ethics and governance requirements,^63^ resources and services can differ which may affect the recruitment process.

## Conclusion

To conclude, an adapted ‘Social Marketing Mix Framework’ incorporating the concepts of ‘emotional labour’, ‘paternalism’ and ‘professional hierarchies and power relationships between clinicians’ is a useful framework for planning and monitoring recruitment activity in a palliative care trial. Those planning trials need to ensure that clinicians, depending on their experience and trial characteristics, have access to training and support to address the ‘emotional labour’ of recruiting to a palliative care trial. The type of training and support required to address this recruitment barrier requires further research.

## Supplemental Material

sj-pdf-1-pmj-10.1177_02692163231197917 – Supplemental material for Health care professional recruitment of patients and family carers to palliative care randomised controlled trials: A qualitative multiple case study Click here for additional data file. Supplemental material, sj-pdf-1-pmj-10.1177_02692163231197917 for Health care professional recruitment of patients and family carers to palliative care randomised controlled trials: A qualitative multiple case study by Lesley Dunleavy, Nancy Preston and Catherine Walshe in Palliative Medicine
