# Supplementary material for: Health care professional recruitment of patients and family carers to palliative care randomised controlled trials: A qualitative multiple case study
Source: Palliat Med. 2023 Sep 27;37(10):1540–53. doi: 10.1177/02692163231197917 (PMC10657513; doi:10.1177/02692163231197917)
Supplement: sj-pdf-1-pmj-10.1177_02692163231197917 – Supplemental material for Health care professional recruitment of patients and family carers to palliative care randomised controlled trials: A qualitative multiple case study [file sj-pdf-1-pmj-10.1177_02692163231197917.pdf]

**Supplementary material for: Health care professional recruitment of patients and family carers to palliative care randomised controlled trials: A qualitative multiple case study**

**Interview Topic Guide**

This is a semi structured interview topic guide for those professionals/staff members involved in recruitment for the palliative care randomised controlled trials selected as 'cases' for this study. The interview topic guide will be iterative and flexible and the topics listed below may not necessarily be covered in order. It will be adapted as appropriate to reflect the individual characteristics of the trial and whether staff from the study coordinating centre is being interviewed or those from a clinical recruitment centre. The interview schedule may be modified and developed further as a result of the interview responses.

- The participant's professional and work experience related to research and palliative care
- Roles and responsibilities, team composition and the characteristics of the setting/s where recruitment activity takes place
- Characteristics of the trial such as inclusion/exclusion criteria guided by the documentation obtained about the trial prior to the interview
- Recruitment procedures for trial participants
- Exploration of phraseology used to discuss the trial with participants
- How well the trial is recruiting or has recruited
- What factors have helped or hindered recruitment to the trial
- Recruitment strategies
- Lessons learnt about recruitment
- Any other issues
